# Trajectory of Tinnitus Distress Across the COVID-19 Pandemic: A Cross-Sectional Analysis of Self-Reported Symptoms

**DOI:** 10.3390/audiolres15050132

**Published:** 2025-10-09

**Authors:** Anusha Yellamsetty, Mika Shin

**Affiliations:** Department of Audiology, San José State University, San José, CA 95192, USA

**Keywords:** tinnitus, COVID-19, vaccination, THI, TRQ, psychological distress, pandemic, audiology

## Abstract

**Purpose:** This study aimed to evaluate retrospectively self-reported changes in tinnitus severity and distress associated with the COVID-19 pandemic and vaccination using validated self-report instruments. It further explored patient-reported onset of tinnitus following vaccination, gender differences in symptom severity, and associations with psychological distress. **Method:** A cross-sectional online survey was completed by 189 adults between December 2023 and April 2024. Participants retrospectively self-reported tinnitus onset and severity before, during, and after the COVID-19 pandemic using the Tinnitus Handicap Inventory (THI) and the Tinnitus Reaction Questionnaire (TRQ). Additional items assessed COVID-19 vaccination history, infection status, and adverse vaccine reactions. Repeated measures ANOVAs and chi-square tests were used to examine retrospectively reported longitudinal changes and group differences. **Results:** Mean THI scores increased significantly over time—before (M = 9.57, SD = 16.00), during (M = 29.97, SD = 32.30), and currently (M = 36.92, SD = 28.04)—with large effect sizes across functional, emotional, and catastrophic subscales (*p* < 0.001). TRQ scores also were reported to rise from before (M = 7.86, SD = 15.29) to during (M = 26.38, SD = 29.58) and remained elevated at the current time (M = 29.79, SD = 24.00), *p* < 0.001. Approximately 25.9% and 27.0% of respondents reported new or worsened tinnitus after the first and second vaccine doses, respectively. No significant gender differences in symptom severity were observed. TRQ severity classification revealed a marked shift, with moderate or greater distress increasing from 5.8% (before) to 35.6% (current). **Conclusions:** Retrospective self-reports indicated that tinnitus burden significantly increased during the COVID-19 pandemic and remained elevated at the time of survey completion. A notable proportion of individuals reported new-onset tinnitus following vaccination, though causality cannot be inferred. These findings highlight the need for continued monitoring and comprehensive care addressing both audiological and psychological components of tinnitus.

## 1. Introduction

The coronavirus disease 2019 (COVID-19) pandemic introduced unprecedented global challenges that extended well beyond the physical health domain, influencing psychological well-being, emotional resilience, and quality of life. In addition to its biological impact, the pandemic triggered widespread psychological distress due to prolonged isolation, uncertainty, and disruption of daily life [1,2]. Taylor [3] emphasized that the psychological footprint of pandemics can exceed the medical footprint, as emotional responses are often more pervasive and longer-lasting than the infection itself. For example, during early lockdowns in 2020, only 2% of a North American sample reported a COVID-19 diagnosis yet 20% experienced elevated anxiety and depression [4].

Stress-related disorders such as post-traumatic stress disorder (PTSD), obsessive–compulsive disorder (OCD), and prolonged grief disorder have all been linked to pandemic-related experiences, including the loss of loved ones, income insecurity, and disruption of social rituals [5]. Xiong et al. [6] reported an increased global burden of mental disorders and suicidality driven by COVID-19-related isolation, financial stress, and uncertainty. These mental health challenges were compounded by reduced access to non-urgent healthcare services, including hearing healthcare and tinnitus management [7].Tinnitus, the perception of sound without an external source, affects a substantial portion of the population and is known to be exacerbated by stress and emotional distress [8,9].Individuals with tinnitus often report worsened symptoms during periods of psychological strain, and the pandemic may have further amplified these effects [10,11]. The multifactorial nature of tinnitus, involving audiological and psychological dimensions, makes it especially susceptible to contextual influences such as those imposed by the pandemic. This complexity is further underscored by tinnitus’s well-documented associations with systemic comorbidities, including cardiovascular, metabolic, and autoimmune conditions [12]. Emerging literature has also identified the COVID-19 vaccine as a potential trigger for new-onset or worsening tinnitus in susceptible individuals. For example, Yellamsetty et al. [13,14,15] and Wang, et al. [16] reported increased tinnitus severity following vaccination, with a notable association between pre-existing metabolic disorders and post-vaccination auditory symptoms. Aydogan et al. [17], Tai et al. [18], and Jiang et al. [6] further support the link between tinnitus and mental health vulnerability during the pandemic. Additional studies [11,15] emphasize the need for ongoing public health surveillance of post-vaccine auditory symptoms, particularly in populations with predisposing risk factors.

Validated instruments such as the Tinnitus Handicap Inventory (THI) [19] and the Tinnitus Reaction Questionnaire [20] provide standardized tools for assessing tinnitus severity and associated psychological distress. These tools were used in the present study to evaluate longitudinal changes in tinnitus perception and reaction from before the pandemic, through its peak, and into the post-pandemic period.

Studying tinnitus symptoms over a longitudinal timeline, capturing data from before, during, and after the COVID-19 pandemic, is essential to understanding the trajectory of auditory and psychological symptom evolution. This approach allows researchers to distinguish between transient and persistent changes in tinnitus severity and identify potential causal patterns associated with vaccination, infection, and psychological stressors. These insights are especially critical for clinicians and policymakers seeking to implement timely interventions and develop robust public health strategies in response to evolving crises.

This study aimed to evaluate self-reported changes in tinnitus severity and distress across timepoints associated with COVID-19 infection and vaccination, using a cross-sectional survey approach. By analyzing self-reported symptoms across multiple timepoints, this research contributes to a growing body of evidence addressing the biopsychosocial burden of tinnitus in the context of a global health crisis.

## 2. Methods

### 2.1. Study Design and Recruitment

This study employed a cross-sectional design to examine the psychological and perceptual effects of COVID-19 and its vaccination on individuals with and without tinnitus. Participants were recruited using a convenience sampling approach [7]. The recruitment strategy included the distribution of a Qualtrics-based online survey via multiple channels: social media platforms (e.g., Facebook, LinkedIn), email outreach to voluntary research registries, and physical flyers posted in audiology clinics in the Bay Area. The survey link was accessible to a national audience across the United States to promote geographic diversity in participation. While this approach facilitated broad outreach, it may have introduced selection bias, as individuals with more severe tinnitus symptoms or heightened interest in COVID-19-related auditory effects may have been more likely to participate. Participant information was self-reported and not independently verified, which we acknowledge as a methodological limitation. Audiometric testing was not conducted as part of this study; thus, analyses were restricted to validated self-report measures rather than objective auditory assessments.

### 2.2. Participants

Eligibility criteria included being at least 18 years of age, residing in the United States, and having a history of COVID-19 vaccination or infection, regardless of tinnitus status. Participants self-reported their demographics, medical history, COVID-19 exposure and vaccination details, and tinnitus experience.

### 2.3. Ethical Considerations

This study received ethical approval from the Institutional Review Board (IRB) at San José State University. Informed consent was obtained electronically prior to survey participation. The consent form outlined the study’s objectives, procedures, confidentiality safeguards, participants’ rights, and contact information for study personnel and the IRB.

### 2.4. Survey Materials

A total of 189 participants completed the online survey between December 2023 and April 2024. All participants were recruited regardless of tinnitus history. The initial dataset included 266 respondents; records with incomplete or missing responses on core demographic and tinnitus-related items were excluded, while partial responders with sufficient information for analysis were retained. To ensure consistency across timepoints, participants who did not report tinnitus at a given time were coded as “no tinnitus”, with corresponding severity/distress scores set to zero. This data cleaning procedure ensured accurate representation of both tinnitus and non-tinnitus cases across all survey periods. The survey consisted of four main components: (1) demographic information; (2) medical history, including pre-existing audiological and systemic conditions; (3) COVID-19 and vaccination history; and (4) two validated self-report instruments assessing tinnitus severity and distress. These instruments included the Tinnitus Handicap Inventory (THI) and the Tinnitus Reaction Questionnaire (TRQ), both of which have been extensively validated in prior tinnitus research. Participants were asked to retrospectively self-report their tinnitus severity and distress at three timepoints before the pandemic, during the pandemic, and at the current (post-vaccination) period. Given the multi-year gap between the onset of the pandemic (2019) and survey completion (2023–2024), these reports are subject to recall bias, which is acknowledged as a study limitation.

### 2.5. Data Analysis Strategy

Statistical analyses were conducted using IBM SPSS Statistics, Version 29.0 (IBM Corp., Armonk, NY, USA) and R version 4.3.1. Descriptive statistics were computed to summarize participant characteristics and survey responses. Differences in tinnitus severity across timepoints (before, during, and current) were analyzed using repeated-measures analysis of variance (ANOVA). Where assumptions of sphericity were violated, Greenhouse–Geisser corrections were applied. Chi-square tests were used to examine group differences in symptom severity across gender and vaccine dose. Significance thresholds were set at *p* < 0.05 for all analyses, and effect sizes (e.g., partial eta squared) were reported where appropriate.

## 3. Results

### 3.1. Descriptive Characteristics of the Sample

A total of 189 participants completed the survey, with 55.6% identifying as female (*n* = 105) and 44.4% as male (*n* = 84). The mean age for male participants was 53.42 years (SD = 16.43), while females had a mean age of 48.19 years (SD = 17.77). An independent samples *t*-test revealed a statistically significant difference in age between male and female participants (*t* (187) = 2.08, *p* = 0.039), with males being significantly older on average.

**Figure 1:**
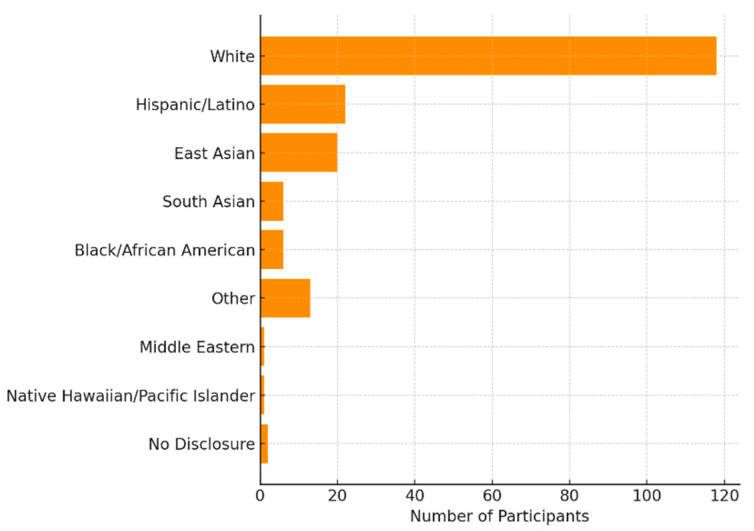
Self-Reported Ethnicity of Study Participants. This horizontal bar chart displays the distribution of ethnic backgrounds among survey participants (*n* = 189). The majority identified as White (62.4%), followed by Hispanic/Latino, East Asian, and smaller proportions from other racial and ethnic groups.

In terms of ethnicity (Figure 1), the majority of participants identified as European American/White (62.4%), followed by Hispanic/Latino (11.6%), East Asian (10.6%), South Asian (3.2%), and Black/African American (3.2%). Smaller proportions identified as Middle Eastern (0.5%), Native Hawaiian/Pacific Islander (0.5%), or Other (6.9%) or selected No Disclosure (1.1%).

### 3.2. Medical History of Participants

**Figure 2:**
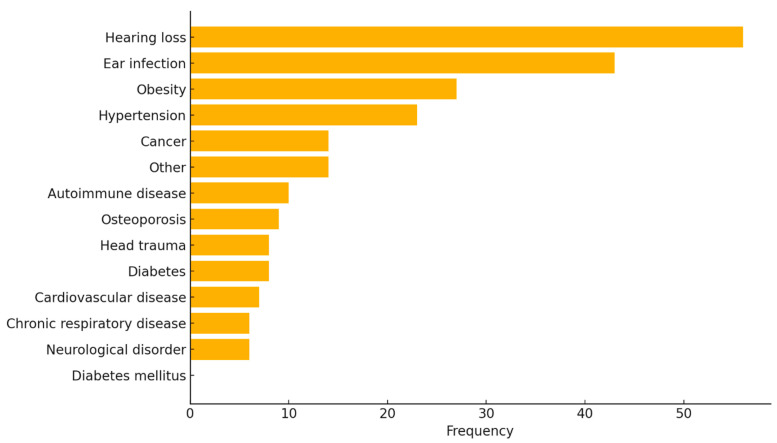
Distribution of Pre-Existing Medical Conditions Reported by Participants. This bar plot illustrates the frequency of self-reported medical conditions among survey participants (*N* = 189). Hearing loss and ear infections were the most reported conditions, followed by obesity and hypertension. No participants reported a diagnosis of diabetes mellitus.

Participants reported a range of pre-existing medical conditions, most commonly hearing loss (29.6%, *n* = 56) and ear infections (22.8%, *n* = 43). Additionally, obesity (14.3%, *n* = 27), hypertension (12.2%, *n* = 23), and cancer or other conditions (each 7.4%, *n* = 14) were also noted. Less frequently reported conditions included autoimmune diseases (5.3%, *n* = 10), osteoporosis (4.8%, *n* = 9), head trauma and diabetes (each 4.2%, *n* = 8), cardiovascular disease (3.7%, *n* = 7), chronic respiratory disease and neurological disorders (each 3.2%, *n* = 6), while diabetes mellitus was not reported (Figure 2). These findings reflect a broad spectrum of comorbidities that may intersect with tinnitus severity and perception in the context of the COVID-19 pandemic.

### 3.3. COVID-19 Vaccination Status

Participants reported their COVID-19 vaccination history across primary doses and booster shots (Table 1). For the first dose, the most common vaccine received was Pfizer-BioNTech (New York, NY, USA) (53.2%, *n* = 100), followed by Moderna (38.3%, *n* = 72), Johnson & Johnson (New Brunswick, NJ, USA) (7.4%, *n* = 14), and Other vaccines (1.1%, *n* = 2). A similar pattern was observed for the second dose, with Pfizer-BioNTech (53.8%, *n* = 91) and Moderna (Cambridge, MA, USA) (43.8%, *n* = 74) being the predominant vaccines, while Johnson & Johnson and Other each accounted for 1.2% (*n* = 2). Among participants who received a first booster, 53.0% (*n* = 70) received Pfizer-BioNTech and 44.7% (*n* = 59) received Moderna, with very few reporting Johnson & Johnson (0.8%, *n* = 1) or another vaccine (1.5%, *n* = 2). The second booster was administered to a smaller group (*n* = 81), with Pfizer-BioNTech and Moderna accounting for 50.6% (*n* = 41) and 43.2% (*n* = 35), respectively. A limited number of participants received Johnson & Johnson (*n* = 2) or other vaccines (*n* = 3). For the third booster, only 50 participants reported vaccine type: 58.0% (*n* = 29) received Pfizer-BioNTech, 40.0% (*n* = 20) received Moderna, and 2.0% (*n* = 1) received a different vaccine. All vaccination data were based on participant self-report and were not independently verified through medical or immunization records.

### 3.4. Adverse Reactions and Symptom Severity Following COVID-19 Vaccination

**Figure 3:**
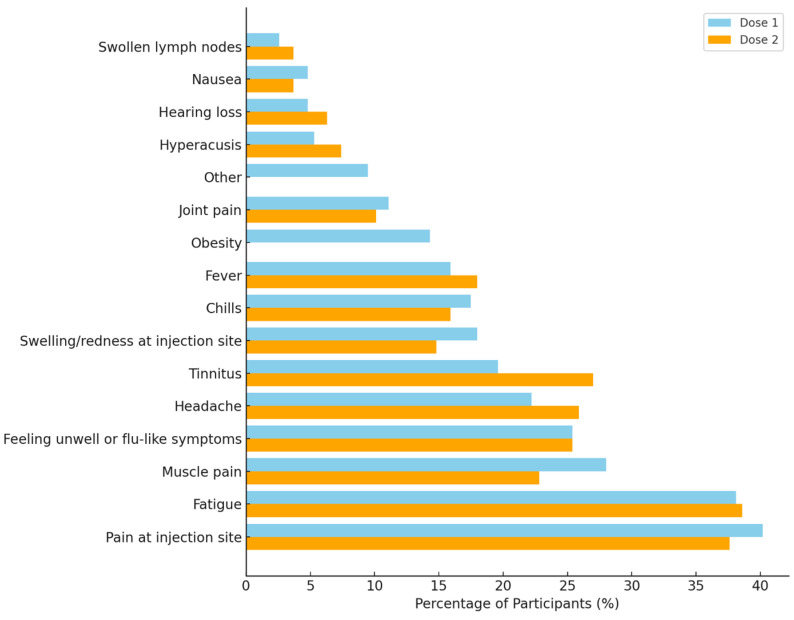
Comparison of self-reported adverse reactions following the first and second doses of COVID-19 vaccination. The most frequently reported symptoms across both doses were pain at the injection site and fatigue, with notable audiological symptoms including tinnitus, hyperacusis, and hearing loss. Percentages reflect the proportion of participants endorsing each symptom.

Participants reported a wide range of adverse reactions following both the first and second doses of the COVID-19 vaccine. After the first dose, the most frequently reported reactions were pain at the injection site (40.2%), fatigue (38.1%), and muscle pain (28.0%). Additional systemic symptoms included feeling unwell or flu-like (25.4%), headache (22.2%), fever (15.9%), and chills (17.5%). Notably, 19.6% of respondents reported tinnitus, along with other audiological symptoms such as hyperacusis (5.3%) and hearing loss (4.8%) (Figure 3).

Reactions following the second dose were comparable, with fatigue (38.6%), pain at the injection site (37.6%), and headache (25.9%) being most commonly reported. A higher proportion of participants reported tinnitus (27.0%) after the second dose compared to the first. Other reactions included muscle pain (22.8%), feeling unwell (25.4%), and fever (18.0%). Audiological symptoms also persisted after the second dose, including hyperacusis (7.4%) and hearing loss (6.3%) (Figure 3).

**Figure 4:**
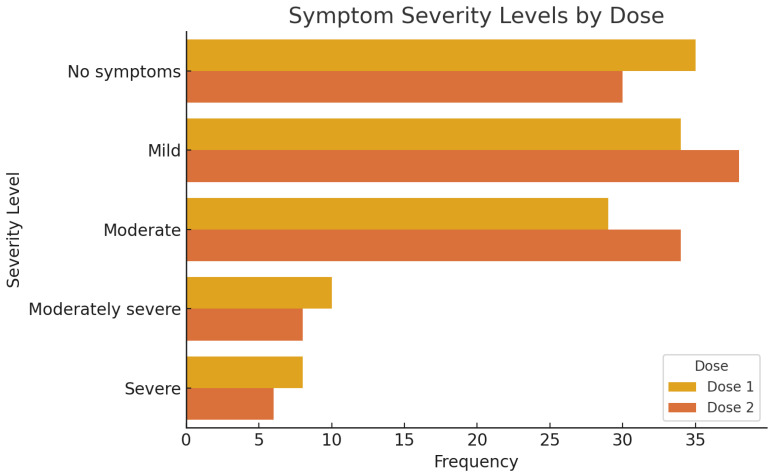
Distribution of symptom severity levels reported following the first and second doses of COVID-19 vaccination. The plot illustrates the number of participants who reported each severity level—No symptoms, Mild, Moderate, Moderately Severe, and Severe—for Dose 1 and Dose 2. While the overall distribution is similar between doses, a slightly higher frequency of moderate symptoms was reported after the second dose.

Symptom severity was evaluated across both doses using a five-level scale (none, mild, moderate, moderately severe, severe), which was chosen arbitrarily for descriptive categorization (Figure 4). After the first dose, 30.2% of participants reported no symptoms, 29.3% reported mild symptoms, 25.0% moderate symptoms, 8.6% moderately severe, and 6.9% reported severe symptoms. For the second dose, 25.9% reported no symptoms, 32.8% mild, 29.3% moderate, 6.9% moderately severe, and 5.2% severe symptoms.

Gender-based comparisons of symptom severity (Table 2) revealed no statistically significant association between gender and symptom burden following either dose. After the first dose, 37.5% of males and 23.3% of females reported no symptoms, whereas 25.0% of males and 48.3% of females reported moderate to severe symptoms. Chi-square analysis showed no significant difference between genders (χ^2^(4) = 3.856, *p* = 0.426). Similarly, after the second dose, 30.4% of males and 21.7% of females reported no symptoms, while 34.8% of males and 51.7% of females reported moderate to severe symptoms. Again, the difference was not statistically significant (χ^2^(4) = 2.298, *p* = 0.682). These findings suggest a consistent adverse event profile across both vaccine doses, with a noteworthy prevalence of audiological symptoms, particularly tinnitus, and similar symptom severity distributions between male and female participants.

### 3.5. Tinnitus Onset Following COVID-19 Vaccination

Out of 189 participants who provided valid responses (Table 3), 49 individuals (25.9%) reported the onset or worsening of tinnitus following the first dose of the COVID-19 vaccine, while 140 participants (74.1%) did not report tinnitus at that timepoint. The “No” category includes both individuals without prior tinnitus and those with tinnitus who did not experience onset or worsening after vaccination. For the second dose, 116 participants responded to the relevant question. Among them, 37 participants (31.9%) reported developing or worsening tinnitus after vaccination, while 79 participants (68.1%) did not report tinnitus onset or change at that timepoint. Notably, 73 participants did not respond to this item, resulting in missing data for a substantial portion of the sample.

### 3.6. COVID-19 Infection Status and Timing Relative to Vaccination

Among all 189 respondents, 34 participants (18.0%) reported being infected with COVID-19 prior to receiving any vaccination. The remaining 155 participants (82.0%) reported no history of infection prior to their initial vaccine dose. In the subgroup who received more than one vaccination, 43 participants (22.8%) reported testing positive for COVID-19 between vaccine doses, while 146 participants (77.2%) did not experience infection in that interval.

### 3.7. COVID-19-Related Hospitalization

Of the 189 total participants, 137 provided responses to the question about COVID-19-related hospitalization. Only one participant (0.7%) reported being hospitalized due to COVID-19, while 136 participants (99.3%) indicated they were not hospitalized. Fifty-two participants (27.5%) did not respond to this question. These findings suggest that severe COVID-19 outcomes requiring hospitalization were rare in this study cohort.

### 3.8. Longitudinal Changes in Tinnitus Handicap Inventory (THI) Scores

Repeated measures ANOVA was conducted to examine changes in tinnitus severity, as measured by the Tinnitus Handicap Inventory (THI), across three time points: before, during, and after the COVID-19 pandemic. Mean THI scores increased progressively from before the pandemic (M = 9.57, SD = 16.00), to during the pandemic (M = 29.97, SD = 32.30), and further to the current point (M = 36.92, SD = 28.04). Mauchly’s test of sphericity indicated a violation of the sphericity assumption (χ^2^(2) = 13.46, *p* = 0.001), so Greenhouse–Geisser correction was applied. The ANOVA revealed a significant main effect of time on THI scores, F(1.84, 270.21) = 69.74 F(1.84, 270.21) = 69.74, F(1.84, 270.21) = 69.74, *p* < 0.001, with a large effect size (partial η^2^ = 0.453). Post hoc pairwise comparisons (LSD-adjusted) showed that THI scores were significantly higher during the pandemic compared to before (*p* < 0.001, mean difference = 20.41), and current scores were significantly higher than both during (*p* < 0.001, mean difference = 6.95) and before the pandemic (*p* < 0.001, mean difference = 27.35). These findings suggest a significant and sustained increase in tinnitus-related handicap over the course of the pandemic, with the highest burden reported at the time of survey.

#### 3.8.1. THI Functional Subscale

**Figure 5:**
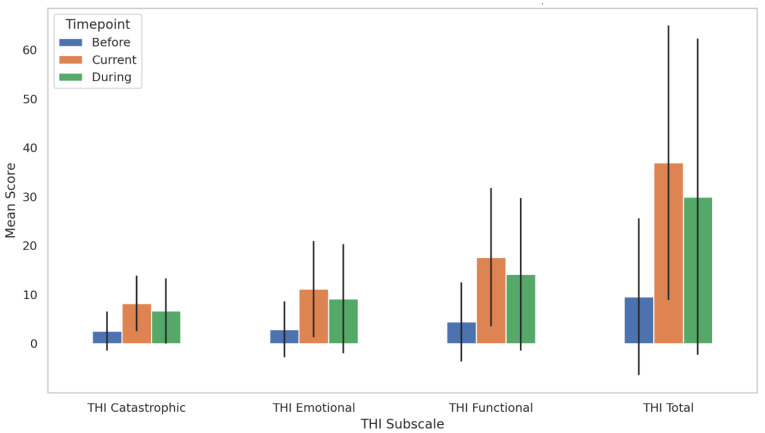
Bar plot illustrates the mean scores of the Tinnitus Handicap Inventory (THI) total and subscale domains (Functional, Emotional, and Catastrophic) across three pandemic timepoints: before, during, and current. Error bars represent standard deviations. Repeated measures ANOVA revealed statistically significant increases in all subscale scores over time (*p* < 0.001), with pairwise comparisons indicating significant differences between each timepoint for all domains. These findings highlight a progressive and persistent increase in tinnitus severity during the COVID-19 pandemic.

Repeated measures ANOVA was conducted to evaluate changes in tinnitus-related functional difficulties (THI Functional subscale) across three time points: before, during, and after the COVID-19 pandemic. Mean functional scores increased from 4.42 (SD = 8.11) before the pandemic to 14.16 (SD = 15.58) during the pandemic and to 17.62 (SD = 14.13) at the current time point (Figure 5). Mauchly’s test of sphericity was violated (W = 0.902, χ^2^(2) = 15.05, *p* < 0.001); therefore, Greenhouse–Geisser corrections were applied. The repeated-measures ANOVA revealed a significant main effect of time, F(1.82, 267.77) = 70.09, F(1.82, 267.77) = 70.09, F(1.82, 267.77) = 70.09, *p* < 0.001, with a large effect size (partial η^2^ = 0.444). Pairwise comparisons (LSD-adjusted) showed that functional scores were significantly higher during the pandemic compared to before (*p* < 0.001, mean difference = 9.74) and continued to rise significantly from during to the current time point (*p* < 0.001, mean difference = 3.46). These results suggest a progressive and statistically significant increase in functional tinnitus burden from pre-pandemic levels through to the present.

#### 3.8.2. THI Emotional Subscale

Repeated measures ANOVA was conducted to assess changes in emotional distress related to tinnitus (THI Emotional Subscale) across three time points: before, during, and after the COVID-19 pandemic. Mean emotional scores increased from 2.88 (SD = 5.70) pre-pandemic to 9.16 (SD = 11.12) during the pandemic and further to 11.11 (SD = 9.87) at the current time point (Figure 5). Mauchly’s test indicated a violation of sphericity (W = 0.897, χ^2^(2) = 15.83, *p* < 0.001); therefore, Greenhouse–Geisser corrections were applied. The ANOVA revealed a significant main effect of time, F(1.81, 265.53) = 52.77, F(1.81, 265.53) = 52.77, F(1.81, 265.53) = 52.77, *p* < 0.001, with a large effect size (partial η^2^ = 0.264). Post hoc pairwise comparisons (LSD-adjusted) indicated significant increases in emotional subscale scores from before to during the pandemic (*p* < 0.001), from before to the current time (*p* < 0.001), and from during to the current time (*p* = 0.006). These findings demonstrate a progressive and statistically significant increase in tinnitus-related emotional distress, with the highest levels reported at the time of the survey.

#### 3.8.3. THI Catastrophic Subscale

Repeated measures ANOVA was conducted to examine changes in catastrophic responses to tinnitus (THI Catastrophic Subscale) across three times: before, during, and after the COVID-19 pandemic. The mean scores increased from 2.55 (SD = 3.97) before the pandemic to 6.65 (SD = 6.63) during the pandemic and further to 8.19 (SD = 5.67) at the current time (Figure 5). Mauchly’s test indicated that the assumption of sphericity was met, χ^2^(2) = 5.70, *p* = 0.058; therefore, sphericity was assumed. The repeated-measures ANOVA revealed a statistically significant main effect of time on catastrophic scores F(2, 294) = 66.11, *p* < 0.001, with a large effect size (partial η^2^ = 0.310). Post hoc pairwise comparisons (LSD-adjusted) indicated that catastrophic scores significantly increased from before to during (*p* < 0.001, mean difference = 4.10), from before to the current time (*p* < 0.001, mean difference = 5.64), and from during to the current time (*p* = 0.001, mean difference = 1.54). These results highlight a statistically significant escalation in catastrophic tinnitus reactions during and after the pandemic period, with the highest scores observed at the time of survey.

### 3.9. Tinnitus Reaction Questionnaire (TRQ)

To assess tinnitus-related distress over time, participants completed the Tinnitus Reaction Questionnaire (TRQ) at three time points: prior to COVID-19 vaccination (“Before”), during the vaccination period (“During”), and at the time of survey completion (“Current”). Descriptive statistics and visual inspection of score distributions revealed meaningful changes across these points.

**Figure 6:**
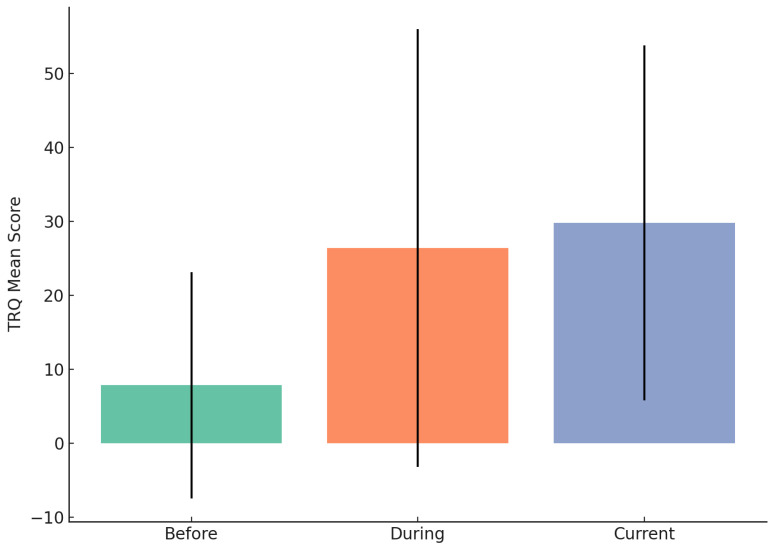
Mean Tinnitus Reaction Questionnaire (TRQ) scores before, during, and after the COVID-19 vaccination period. Error bars represent standard deviations. A notable increase in tinnitus-related distress is observed from the pre-vaccination period to the cur.

The mean TRQ score prior to vaccination was 9.96 (SD = 15.01), indicating minimal tinnitus-related distress for most participants. During the vaccination period, TRQ scores increased significantly, with a mean of 29.74 (SD = 28.46). This marked increase reflects a subset of participants experiencing the onset or exacerbation of tinnitus-related symptoms during this period. At the time of the survey (Current), the mean TRQ score remained elevated at 29.75 (SD = 26.97), which suggested persistent distress in a subgroup of participants (Figure 6). A repeated-measures ANOVA confirmed a statistically significant effect of time on TRQ scores, F(2, 212) = 65.74, *p* < 0.001, with pairwise comparisons indicating significant increases from Before to During (*p* < 0.001) and Before to Current (*p* < 0.001) but no significant change from During to Current (*p* = 0.995). This pattern suggests that tinnitus distress worsened during the vaccination period and remained elevated at the time of data collection.

These findings provide empirical support for a potential link between the COVID-19 vaccination period and increased tinnitus-related distress in a subset of individuals.

#### TRQ Severity Levels Across Timepoints

The distribution of tinnitus-related distress, as assessed by the Tinnitus Reaction Questionnaire (TRQ) severity levels, (Table 4) showed notable changes across the three assessed timepoints: before, during, and at the time of survey completion (“current”). Prior to the COVID-19 pandemic, the vast majority of participants (81.3%, *n* = 87) fell within the “No/Slight” severity category, indicating minimal tinnitus-related distress. Only small proportions reported mild (13.1%, *n* = 14), moderate (3.7%, *n* = 4), severe (0.9%, *n* = 1), or very severe distress (0.9%, *n* = 1).

During the pandemic, a shift toward greater severity was observed. The proportion of participants in the “No/Slight” category decreased to 51.4% (*n* = 55), while increases were seen in mild (19.6%, *n* = 21), moderate (11.2%, *n* = 12), severe (6.5%, *n* = 7), and very severe (11.2%, *n* = 12) categories.

At the time of the survey (“current”), the proportion in the “No/Slight” category further declined to 33.6% (*n* = 36). The number of participants reporting mild (33.6%, *n* = 36) and moderate (16.8%, *n* = 18) severity increased, and severe (10.3%, *n* = 11) and very severe (5.6%, *n* = 6) cases remained notable. These results indicate a progressive increase in tinnitus-related distress throughout the pandemic period, with a substantial shift from lower to higher severity categories.

## 4. Discussion

This study offers a comprehensive examination of the longitudinal patterns of the effects of the COVID-19 pandemic and subsequent vaccination on tinnitus-related distress and severity, as retrospectively self-reported and measured using the Tinnitus Handicap Inventory (THI) and Tinnitus Reaction Questionnaire (TRQ). Our results indicate a significant and sustained increase in tinnitus burden from pre-pandemic to the present timepoint, with all THI subscales, functional, emotional, and catastrophic, demonstrating progressive and statistically significant elevations. Repeated measures ANOVAs revealed large effect sizes, highlighting the clinical relevance of these shifts.

Changes in TRQ severity classifications were particularly striking: the proportion of participants experiencing moderate or greater tinnitus-related distress tripled from the baseline to the current timepoint. These findings are consistent with prior studies reporting an exacerbation of tinnitus symptoms during the pandemic [7,11], likely reflect participants’ retrospective accounts of compounded effects of psychosocial stress, isolation, and disruptions to routine healthcare access.

We also identified notable patterns related to COVID-19 vaccination. New-onset or worsening tinnitus temporally associated with vaccination was reported by 25.9% of participants after the first dose and 31.9% after the second dose. These rates are consistent with the emerging literature [13,14,15,16] and underscore the importance of continued monitoring. Importantly, vaccination history and tinnitus changes were self-reported, and causality cannot be inferred from this observational design. In addition, “No” responses included both participants without tinnitus history and those with pre-existing tinnitus who did not experience onset or worsening, highlighting the heterogeneity of symptom trajectories in this cohort. The temporal associations, particularly among individuals with preexisting metabolic conditions [16], warrant further investigation into possible biological and psychological mechanisms but should be interpreted cautiously.

Interestingly, gender-based analyses did not reveal statistically significant differences in symptom severity or trajectory post-vaccination. This finding aligns with the work of [21], who similarly found no sex-based differences in auditory outcomes in post-viral populations, suggesting that gender may not be a key moderator in vaccination-related tinnitus onset or distress.

The observed increase in TRQ scores over time lends support to cognitive–emotional models of tinnitus, such as the framework proposed by Tyler et al. [22], which emphasizes the roles of anxiety, attention, and rumination in tinnitus-related distress. These findings reaffirm the value of the biopsychosocial model in clinical tinnitus management and highlight the importance of integrating mental health screening and psychological support into standard audiological care.

Moreover, our results contribute to the growing body of literature identifying strong associations between tinnitus and mental health challenges, particularly during global crises. Jiang et al. [23] recently demonstrated robust links between tinnitus severity and depression, anxiety, stress, and suicidal ideation. Their findings of altered limbic activity and dysregulation of the hypothalamic–pituitary–adrenal (HPA) axis provide a plausible neurobiological basis for the sustained increases in tinnitus distress observed in our cohort. Recent work further supports this perspective, emphasizing that pandemic-related stress may act as a potent trigger or amplifier of tinnitus through psychological and neurobiological pathways, rather than through direct alterations of auditory physiology [24]. Compared to prior investigations that have primarily examined either COVID-19 infection [7,25] or vaccination-related tinnitus in isolation e.g., [13,14,15,26], our study is distinctive in that it used validated instruments (THI, TRQ) [19,20] to capture self-reported tinnitus severity across three distinct timepoints (pre-pandemic, pandemic, and current/vaccination). This design provides a broader perspective on both psychosocial and vaccine-related influences, allowing for a more comprehensive characterization of tinnitus trajectories. This divergence underscores the need for future research to distinguish stress-mediated mechanisms from peripheral auditory effects when examining tinnitus trajectories in pandemic and post-vaccination contexts.

## 5. Conclusions

This study provides retrospective, self-reported insights into the sustained patterns of evidence of the sustained and multifactorial impact of the COVID-19 pandemic and vaccination on tinnitus severity and distress. The observed increases in THI and TRQ scores from pre-pandemic to current timepoints reflect participants’ retrospective reports and underscore the importance of addressing both the auditory and psychological dimensions of tinnitus within clinical care. The substantial proportion of participants reporting new-onset or worsened tinnitus symptoms following COVID-19 vaccination further emphasizes the need for ongoing monitoring and clear, evidence-based communication with patients.

Our findings support the biopsychosocial model of tinnitus, highlighting how stress, isolation, and reduced healthcare access may amplify tinnitus-related burden. Importantly, they reinforce the need for interdisciplinary approaches that integrate audiologic management with mental health screening and psychological support. However, as these findings are based on retrospective self-reports, they should be interpreted as associative rather than causative.

## 6. Limitations

Several limitations should be acknowledged. First, the study’s observational design precludes causal inference between COVID-19 vaccination and tinnitus onset or exacerbation. Second, all data were self-reported, which may introduce recall bias or reporting inaccuracies, particularly regarding symptom onset and severity. Third, the lack of audiometric or neurophysiological data limits our ability to objectively verify changes in hearing or tinnitus perception. Additionally, while the sample was demographically diverse, it may not be representative of the broader population, potentially limiting generalizability. Finally, mental health variables such as anxiety or depression were not directly measured and should be considered in future research.

## 7. Future Directions

Future studies should aim to clarify the biological and neuropsychological mechanisms linking vaccination, pandemic-related stress, and tinnitus. This includes examining the role of pre-existing metabolic or psychological vulnerabilities and potential involvement of the hypothalamic–pituitary–adrenal (HPA) axis. Longitudinal cohort studies incorporating objective audiological assessments, biomarkers of stress and inflammation, and validated mental health measures are warranted. Additionally, randomized controlled trials evaluating integrated interventions such as tinnitus retraining therapy combined with cognitive–behavioral or stress-reduction strategies may inform best practices in managing tinnitus in post-pandemic clinical settings.

## Figures and Tables

**Table 1:** COVID-19 Vaccine Type by Dose Reported by Participants. This table summarizes the distribution of vaccine types received across up to five COVID-19 vaccine doses. Pfizer-BioNTech was the most frequently reported vaccine for all doses, followed by Moderna. Johnson & Johnson and other vaccine types were reported less frequently, particularly for booster doses.

Dose	Pfizer-BioNTech	Moderna	Johnson & Johnson	Other
First Dose	100	72	14	2
Second Dose	91	74	2	2
1st Booster	70	59	1	2
2nd Booster	41	35	2	3
3rd Booster	29	20	0	1

**Table 2:** Symptom severity stratified by dose and gender.

Severity Level	Dose 1 Male	Dose 1 Female	Dose 2 Male	Dose 2 Female
No symptoms	21	14	17	13
Mild	17	17	16	22
Moderate	11	18	13	21
Moderately severe	4	6	2	6
Severe	3	5	2	4

**Table 3:** Summary of key binary outcomes reported by participants. Values indicate the number and percentage of participants who responded “Yes” or “No” to each item. For tinnitus outcomes, “No” reflects participants who did not report tinnitus at the specified timepoint, which includes both individuals without prior tinnitus and those who did not experience onset or worsening. Percentages for tinnitus after the second dose are calculated from the 116 participants who provided responses.

Variable	Yes (*n*, %)	No (*n*, %)
Tinnitus after first dose	49 (25.9%)	140 (74.1%)
Tinnitus after second dose	37 (19.6%) *	79 (41.8%) *
COVID-19 infection prior to vaccination	34 (18.0%)	155 (82.0%)
COVID-19 positive between vaccine doses	43 (22.8%)	146 (77.2%)

Note: * *p* < 0.05 (statistically significant).

**Table 4:** Distribution of Tinnitus Reaction Questionnaire (TRQ) Severity Levels Across Three Timepoints: Before, During, and Current.

TRQ Severity Levels	Before	During	Current
No/Slight	87	55	36
Mild	14	21	36
Moderate	4	12	18
Severe	1	7	11
Very Severe	1	12	6

## Data Availability

The data presented in this study are available on request from the corresponding author. The data is not publicly available due to privacy and ethical restrictions.
